# 1290. Ceftazidime-Avibactam Resistance Report in a Third Level Hospital in Mexico City

**DOI:** 10.1093/ofid/ofab466.1482

**Published:** 2021-12-04

**Authors:** Aaron Molina, Abigail Salinas-Hernandez, Alejandro Olmedo-Reneaum

**Affiliations:** 1 ISSSTE, Mexico City, Distrito Federal, Mexico; 2 Instituto de Seguridad y Servicios Sociales de los Trabajadores del Estado, Ciudad de Mexico, Distrito Federal, Mexico; 3 IM resident @ Médica Sur, Mexico City, Distrito Federal, Mexico

## Abstract

**Background:**

The surge of resistant Gram-negative organisms has been worrying infectious disease physicians and physicians in general because of the lack of a large number of antibiotics to which these organisms remain susceptible. Ceftazidime-Avibactam (CAZ-AVI) is a drug approved by the FDA to treat complicated urinary tract infections (cUTI), complicated intra-abdominal infections (cIAIs) in combination with metronidazole, and recently for the treatment of nosocomial pneumonia. Worldwide resistance rates of Enterobacteriaceae to CAZ-AVI have been reported below 2.6%, and 4-8% for *Pseudomonas aeruginosa*. The FDA, CLSI, and EUCAST assigned the clinical breakpoints of susceptibility: MIC < /=8 mg/liter susceptible, and >/=8mg/liter, resistant. In Mexico, CAZ-AVI was approved in 2018, and its cost is very high compared to other antimicrobials, so its use is limited in very specific cases. The resistance rates to this antibiotic in the Mexican population remain largely unknown.

**Methods:**

We tested 106 specimens for susceptibility to ceftazidime-avibactam using the disk Kirby-Bauer method. The inhibition zone diameter was determined in all cases and we considered the organism susceptible when the inhibition zone diameter was >=21 mm, and resistant with an inhibition zone diameter < = 20 mm.

**Results:**

We found 5 specimens (4.71%) resistant to ceftazidime-avibactam, corresponding to *E. coli* (3) and *P. aeruginosa* (2). Two of these were also resistant to colistin, and 4 to meropenem. All carbapenem-resistant isolates harbored Metallo-beta-lactamases genes, for *E. coli* was NDM gen, and for *P. aeruginosa* the VIM gene(GeneXpert® Cepheid).

**Conclusion:**

The ceftazidime-avibactam resistance among Gram-negative bacteria in our study is similar to the one reported in other international studies. We need more studies in our population to know the nationwide resistance to this antibiotic.

**Disclosures:**

**All Authors**: No reported disclosures

